# Progesterone Prevents Traumatic Brain Injury-Induced Intestinal Nuclear Factor kappa B Activation and Proinflammatory Cytokines Expression in Male Rats

**DOI:** 10.1155/2007/93431

**Published:** 2007-08-20

**Authors:** Gang Chen, Jinxin Shi, Yasuo Ding, Hongxia Yin, Chunhua Hang

**Affiliations:** Department of Neurosurgery, Jinling Hospital, School of Medicine, Nanjing University, Nanjing, Jiangsu Province 210002, China

## Abstract

We have previously shown that traumatic brain injury (TBI) can induce an upregulation of nuclear factor kappa B (NF-κB) and proinflammatory cytokines in the gut, which play an important role in the pathogenesis of acute gut mucosal injury mediated by inflammation. In this work, we investigated whether progesterone administration modulated intestinal NF-κB activity and proinflammatory cytokines expression after TBI in male rats. As a result, we found that administration of progesterone following TBI could decrease NF-κB binding activity, NF-κB p65 protein expression, and concentrations of interleukin-1β (IL-1β), and tumor necrosis factor-α (TNF-α) in the gut. TBI-induced damages of gut structure were ameliorated after progesterone injections. The results of the present study suggest that the therapeutic benefit of post-TBI progesterone injections might be due to its inhibitory effects on intestinal NF-κB activation and proinflammatory cytokines expression.

## 1. INTRODUCTION

The importance of the intestinal mucosa in the inflammatory and metabolic responses to sepsis, trauma, and other critical illnesses is increasingly recognized. Major trauma and shock may initiate a cascade of intestinal events such as intestinal cytokine overproduction [1], increased intestinal permeability [2], and translocation of intestinal bacteria and endotoxins [3]. These events may not only influence the intestinal mucosa itself, but also may affect the function and integrity of remote organs and tissues, leading to systemic inflammatory response syndrome (SIRS) and multiple organ dysfunction syndrome (MODS). In our previous study [4], it has been demonstrated that traumatic brain injury (TBI) can induce marked damage of intestinal mucosa structure including the shedding of epithelial cells, fracture of villi, focal ulceration, fusion of adjacent villi, dilation of the central chyle duct, mucosal atrophy, and disruption of the tight junction between enterocytes. Afterwards, we found that inflammatory response mediated by increased nuclear factor kappa B (NF-κB) and proinflammatory cytokines played a crucial pathological role in acute gut mucosal injury following TBI [5]. However, little information was found in the literatures about any effective treatment that could modulate intestinal NF-κB activation and proinflammatory cytokines production after TBI.

Since its identification as an inducible activator of gene transcription from the intronic κ light chain, the nuclear transcription factor, NF-κB, has been implicated in the regulation of a growing number of gene products that contribute to the pathogenesis of numerous clinical disorders [6]. NF-κB functions as homo- or heterodimers of the Rel family of proteins, which includes p50, p65, c-Rel, p52, and RelB [7]. The most common combination of subunits is a hererodimer of the p50 and p65 proteins. NF-κB normally exists in the cytoplasm of cells bound by a member of the inhibitor kappa B (I-κB) protein family. NF-κB activation by inducers, such as IL-1β [7, 8] and TNF-α [9], leads to phosphorylation and degration of the I-κB protein allowing NF-κB translocation to the nucleus where it can then bind to specific sites within the promoter sequence of target genes whose products, such as proinflammatory cytokines, inducible nitric oxide synthase (iNOS), cyclooxygenase-2 (COX-2), and acute phase proteins, are critical to inflammatory processes [10, 11]. This kind of positive feedback in the interaction of NF-κB with proinflammatory cytokines may occur through extracellular mechanisms, which has been confirmed by several studies [12, 13]. NF-κB activation enhances the transcription of TNF-α and IL-1β, and both of these cytokines are known to in turn activate NF-κB [14]. The positive feedback is believed to serve to amplify inflammatory signals.

Several experimental studies have demonstrated that progesterone played neuroprotective roles in TBI including reducing cerebral edema, preventing neuronal loss, and improving functional outcomes [15, 16]. One of the specific mechanisms by which progesterone acts to promote morphological and functional recovery is via inhibiting the TBI-induced cerebral NF-κB activation and proinflammatory cytokines up-regulation [17, 18]. Nevertheless till now, no study was found in the literature to investigate the effects of progesterone on intestinal expression of NF-κB and proinflammatory cytokines. In the present study, we investigated the influence of progesterone administration on NF-κB activity and proinflammatory cytokines expression in the small bowl following TBI in male rats.

## 2. MATERIALS AND METHODS

### 2.1. Animals

Male Wistar rats (300 to 350 g) were purchased from Animal Center of Chinese Academy of Sciences, Shanghai, China. The rats were housed in temperature and humidity controlled animal quarters with a 12-hour light/dark cycle. All procedures were approved by the Institutional Animal Care Committee and were in accordance with the guidelines of the National Institutes of Health on the care and use of animals.

### 2.2. Experiment protocol

Rat model of cortical contusion trauma: following intraperitoneal anesthesia with urethane (1000 mg/kg), animal head was fixed in the stereotactic frame. A right parietal craniotomy (diameter 5 mm) was drilled under aseptic conditions 1 mm posterior and 2 mm lateral to the bregma. We used a modification of the Feeney's weight-drop model [19] in which a freefalling weight onto the exposed intact cranial dura produced a standardized parietal contusion by letting a steel rod weighing 40 g with a flat end diameter of 4 mm fall onto a piston resting on the dura from a height of 25 cm. The piston was allowed to compress the tissue a maximum of 5 mm. After operation procedures, the rats were then returned to their cages and the room temperature was kept at 23±1°C. Heart rate, arterial blood pressure and rectal temperature were monitored, and the rectal temperature was kept at 37±0.5°C, by using physical cooling (ice bag) when required, throughout experiments. Sham-operated rats were anesthetized, mounted in the stereotaxic apparatus, and had their scalps cut and sutured but were not trephinated.

The experimental groups consisted of sham + vehicle (SV; n=6), lesion + vehicle (LV; n=6), and lesion + progesterone (LP; n=6). Rats of LP group received injections of 16 mg/kg progesterone (4-Pregnene-3, 20-dione, Sigma-aldrich Inc., St. Louis, MO, USA) at 1 and 6 hours and 1, 2, 3, 4, and 5 days after the surgery (intraperitoneally for the first and subcutaneously for the remaining six). Rats of SV and LV groups received equal volumes of vehicle (2-hydroxypropyl-β-cyclodextrin, Sigma-aldrich Inc.) [20, 21]. The animals were decapitated 5 days after injury for tissue assays. A 3-cm segment of the mid-ileum was taken and flushed with ice-cold saline. Half of it was immersed in neutral-buffered formalin for immunohistochemical assay and histopathological study. The other was stored in liquid nitrogen immediately for electrophoretic mobility shift assay (EMSA) and enzyme-linked immunosorbent assay (ELISA).

### 2.3. Nuclear protein extract and EMSA

Nuclear protein was extracted and quantified as described [5]. EMSA was performed using a commercial kit (Gel Shift Assay System; Promega, Madison, WI, USA) following the methods in our laboratory. The NF-κB oligonucleotide probe (5′-AGTTGAGGGGACTTTCCCAGGC-3′) was end-labeled with [γ-^32^P] ATP (Free Biotech, Beijing, China). EMSA was performed according to our previous study [5]. After electrophoresis, the gel was transferred to Whatman filter paper, vacuum dried and exposed to Kodak XAR-5 film overnight. Levels of NF-κB DNA binding activity were quantified by scanning the developed Kodak XAR-5 film with a computer-assisted, linear scanning densitometer in transparent mode (Hoefer Scientific Instruments, San Francisco, CA, USA). Data were expressed as arbitrary densitometry units (ADU) obtained from the densitometric scans.

### 2.4. Detection of NF-κB p65 and p50 expression in ileum tissue

Immunohistochemical studies were conducted on formalin-fixed, paraffin-embedded sections. The rabit-anti-rat monoclonal antibodies of NF-κB p65 and p50 (both diluted 1 : 100, Santa Cruz Biotechnology, Inc., CA, USA) were used. For immunohistochemistry, sections were incubated in phosphate-buffered saline (PBS) with 5% normal horse serum and 0.3% Triton X-100 for 1 hour at room temperature. Sections were washed three times with PBS and incubated with primary antibody for 2 hours at room temperature. After washing with PBS, sections were incubated with biotinylated second antibodies for 1 hour at room temperature. Sections incubated in the absence of primary antibody were used as negative controls. Microscopy of the immunohistochemically stained tissue sections was performed by an experienced pathologist blinded to the experimental condition. Therfore, evaluation of sections was undertaken by assessing the intensity of staining (5 grades). “0” indicates that there were no detectable positive cells; “1” indicates very low density of positive cells; “2” indicates a moderate density of positive cells; “3” indicates the higher, but not maximal density of positive cells; and “4” indicates the highest density of positive cells.

### 2.5. Detection of IL-1β and TNF-α in ileum tissue

The frozen ileum tissue was homogenized with a glass homogenizer in 1 ml of buffer containing 1 mmol/L of PMSF, 1 mg/L of pepstatin A, 1 mg/L of aprotinin, and 1 mg/L of leupeptin in PBS solution (pH 7.2) and centrifuged at 12,000 g. for 20 minutes at 4°C. The intestinal levels of inflammatory mediators were quantified using specific ELISA kits for rats according to the manufacturers' instructions (TNF-α from Diaclone Research, France; IL-1β from Biosource Europe SA, Belgium). The cytokine contents in the ileum tissue were expressed as nanograms of cytokines per gram of protein.

### 2.6. Histopathological examination

The neutral-buffered formalin-fixed ileum was embedded in paraffin, sectioned at 4 *μ*m thickness with a microtome and stained with hematoxylin and eosin (H and E). The sections were examined under light microscope. Villous height, diameter of middle villous segment, and crypt depth in all tissues were determined using the HPIAS-1000 colorful image analysis system (Champion Image Engineering Company, Wuhan, China) [4]. Villous surface area was calculated according to the following formula: surface area = πdh (d, diameter; h, villous height). At least 10 well-oriented crypt-villous units per small intestinal sample were measured and averaged by a pathologist who was blind to animal groups.

### 2.7. Statistical analysis

The data were presented as mean ± SD. SPSS 12.0 was used for statistical analysis of the data. The comparisons of NF-κB p65 and p50 grades among various groups were conducted by using Mann-Whitney U test. The other measurements were analyzed by one-way ANOVA, followed by Tukey *post hoc* test. Statistical significance was inferred at P⁢< .05.

## 3. RESULTS

### 3.1. General observations

Rats in SV group usually recover consciousness during 12 hours after anesthesia and sham operation, whereas rats in both LV and LP groups were temporally unconscious for 48 hours post-injury and recovered almost normal eating behavior from 72 hours after cortical contusion trauma. During the period of unconsciousness, the rats were given 3 ml of water per 6 hours by tube feeding. After the right parietal cortical contusion, the rats failed to fully extend the left forepaw. The mean arterial blood pressure values and body temperature were within the normal physiological range for all animals (data not shown). The mean baseline weight of the rats in the three groups did not significantly differ before injury.

### 3.2. NF-κB binding activity in the ileum tissue

**Figure 1:**
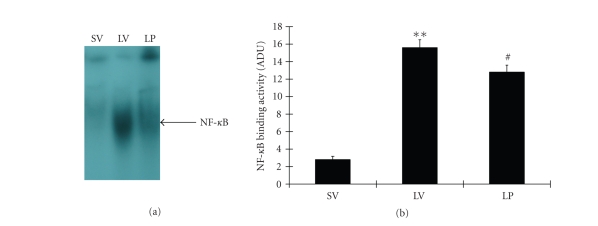
NF-κB activity in the ileum tissue in SV group (sham + vehicle, n⁢= 6), LV group (lesion + vehicle, n⁢= 6), and LP group (lesion + progesterone, n⁢= 6). (Upper) EMSA autoradiography of NF-κB DNA binding. (Bottom) Levels of NF-κB DNA binding activity quantified by computer-assisted densitometric scanning and expressed as an arbitrary densitometric units (ADU). As compared with SV group, NF-κB binding activity measured by EMSA was significantly increased in LV group. Compared to LV group, progesterone significantly suppressed NF-κB activation in LP group. **P⁢< .01 versus SV group, ^#^ P⁢< .05 versus LV group.

EMSA autoradiography of NF-κB DNA binding activity of the ileum tissue samples was shown in Figure 1. Low NF-κB binding activity (weak EMSA autoradiography) was found in the SV group. Compared with SV group, NF-κB binding activity in the small bowl was significantly increased (P⁢< .01) in LV group. In LP group, intestinal NF-κB binding activity was significantly down-regulated (P⁢< .05) after progesterone injections.

### 3.3. Expression of NF-κB p65 and p50 in ileum tissue

**Figure 2:**
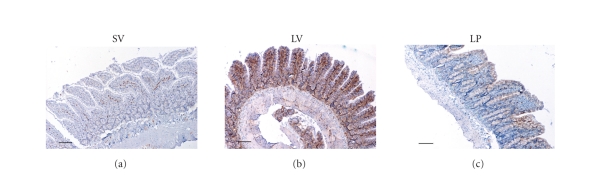
Expression of NF-κB p65 in the small intestines in SV group (sham + vehicle, n⁢= 6), LV group (lesion + vehicle, n⁢= 6), and LP group (lesion + progesterone, n⁢= 6). (a) Rats of SV group showing low NF-κB p65 immunoreactivity; (b) Rats of LV group showing increased NF-κB p65 immunoreactivity stained as brown; (c) Rats of LP group showing less NF-κB p65 immunoreactivity than LV group (scar bar, 100 *μ*m).

**Figure 3:**
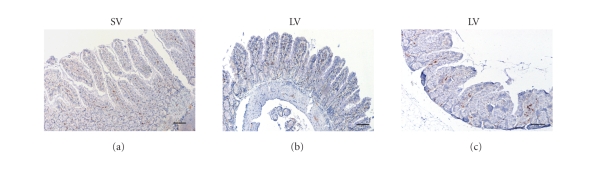
Expression of NF-κB p50 in the small intestines in SV group (sham + vehicle, n⁢= 6), LV group (lesion + vehicle, n=6), and LP group (lesion + progesterone, n⁢= 6). Very low NF-κB p50 immunoreactivity was shown in the ileum tissue of the three groups (scar bar, 100 *μ*m).

**Figure 4:**
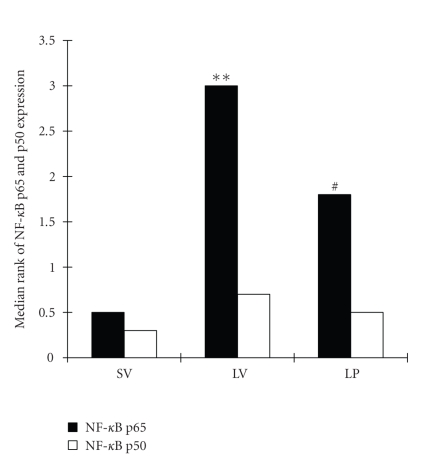
NF-κB p65 and p50 grades in the small intestines in SV group (sham + vehicle, n⁢= 6), LV group (lesion + vehicle, n⁢= 6), and LP group (lesion + progesterone, n⁢= 6). Administration of progesterone remarkably inhibited TBI-induced up-regulation of NF-κB p65 expression in rat ileum tissue. The NF-κB p50 expression in ileum was not significantly different among groups of SV, LV and LP. **P⁢< .01 versus SV group; ^#^ P⁢< .05 versus LV group.

The immunohistochemical assay showed lower NF-κB p65 immunoreactivity in the lamina propia of the mucosa and the interstitial region in SV group (see Figure 2). Compared to SV group, NF-κB p65 was significantly up-regulated (P⁢< .01) in villous interstitium and lamina propria following TBI (see Figures 2 and 4). In LP group, the number of NF-κB p65 immunoreactivity was significantly decreased (P⁢< .05) (see Figures 2 and 4). The results showed that progesterone administration could significantly down-regulate the NF-κB p65 immunoreactivity in rat intestine following TBI (see Figures 2 and 4). The NF-κB p50 expression in ileum was not significantly different among groups of SV, LV, and LP, in which there were very low NF-κB p50 immunoreactivity in the villous interstitium and lamina propria (see Figures 3 and 4).

### 3.4. Intestinal levels of IL-1β and TNF-α

**Figure 5:**
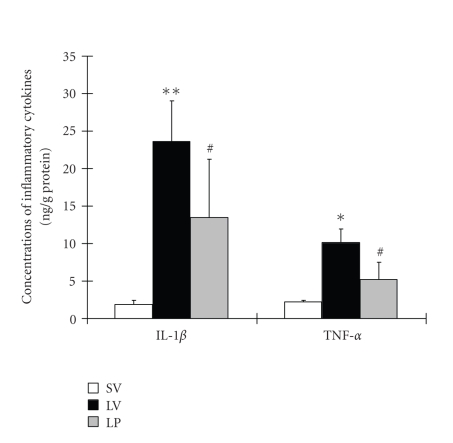
Changes of inflammatory mediators in the small intestines as determined by ELISA in SV group (sham + vehicle, n⁢= 6), LV group (lesion + vehicle, n⁢= 6), and LP group (lesion + progesterone, n⁢= 6). TBI could induce the significantly increased concentrations of IL-1β and TNF-α in rat ileum tissue. In LP group, the intestinal concentrations of IL-1β and TNF-α were markedly down-regulated as compared with that of LV group. **P⁢< .01 and *P⁢< .05 versus SV group; ^#^ P⁢< .05 versus LV group.

Concentrations of IL-1β and TNF-α were low in the rat gut of SV group (1.85±0.56 and 2.23±0.21 ng/g protein, resp.) (see Figure 5). Compared with SV group, intestinal levels of the two inflammatory cytokines were greatly induced after TBI. As shown in Figure 5, progesterone administration after TBI could lead to significantly decreased IL-1β and TNF-α concentrations (P⁢< .05) in rat ileum tissue.

### 3.5. Histomorphometric studies

Villous height, crypt depth, and villous surface area were determined as specific indices for the evaluation of mucosal damages. The parameters are shown in Table 1. Quantitative analyses of the villi demonstrated that the villous height and crypt depth were significantly decreased in LV group as compared with SV group (P<.05). In LP group, villous height and crypt depth were increased significantly as compared with LV group (P⁢< .01). Although villous surface area was not decreased significantly in LV group, the intestinal villous surface area was increased significantly after progesterone injections (P<.05).

## 4. DISCUSSION

The major changes of gastrointestinal function after TBI may be classified into four aspects: stress ulcer, gut motility dysfunction, disruption of gut barrier, and alterations of mucosal absorptive function. We previously reported that traumatic brain injury could lead to significant damages of gut structure and impairment of barrier function [4]. The intestinal inflammatory response following TBI is characterized by intestinal recruitments of neutrophils and monocytes through up-regulating of NF-κB and releasing proinflammatory cytokines, which can lead to gut mucosal injury [5]. In this present study, we found that progesterone administration could decrease the NF-κB binding activity, NF-κB p65 protein expression and concentrations of IL-1β and TNF-α in the small bowl following TBI. It was also demonstrated that TBI-induced damages of gut structure were ameliorated when treated with progesterone.

Steroids are known regulators of NF-κB activity [22, 23]. Previous studies of the anti-inflammatory properties of progesterone have been largely characterized in female reproductive tissue, where progesterone down-regulates COX-2 via NF-κB inhibition [24, 25]. Progesterone has been reported to inhibit NF-κB-mediated transcription in two ways. The better-characterized route is by promoting the production of the endogenous inhibitor of NF-κB (I-κB) [26]. Recent studies show that, when progesterone is present, the progesterone receptor forms a heterodimer with NF-κB p65 and in turn inhibits binding of either transcription factor to its binding site [23]. In this present study, we found the NF-κB binding activity in the nuclear protein was induced by TBI and repressed by progesterone administration. Moreover, we proved that the main up-regulated part of NF-κB was the subunit p65, but not p50. After progesterone injections, intestinal level of NF-κB p65 was decreased significantly. Although the current data have demonstrated that progesterone administration suppressed the TBI-induced intestinal NF-κB up-regulation, changes about the expression of progesterone receptor (PR) and the whole signaling pathway in the gut remain unknown.

In the research regarding progesterone and NF-κB, Pettus et al. [18] used Western blot techniques to analyze the effect of progesterone on cerebral expression of NF-κB. They found NF-κB p65 was increased in all injured animals and decreased by progesterone treatment in comparison to vehicle-treated animals. Measures of NF-κB p50 showed no change after injury or progesterone treatment. Horie et al. [27] investigated the influence of progesterone on NF-κB activation in vitro and suggested that progesterone could reduce TNF-α-induced NF-κB activation in endometriotic stromal cells. Another in vitro study [28] demonstrated that progesterone acted through multiple mechanisms to inhibit NF-κB. First, there was direct inhibition of NF-κB binding to DNA by PR which did not depend upon the presence of ligand and, presumably, was not related to the transcriptional activity of PR. Second, through the isoform progesterone receptor B (PRB), progesterone inhibited the transcription of tumor necrosis factor-α related apoptosis inducing ligand (TRAIL, an activator of NF-κB), and through PRA + PRB, progesterone inhibited the transcription of its receptor, TRAILR2. Finally, progesterone induced the transcription of A20 and ABIN-2 through PRB, resulting in the inactivation of free NF-κB in the cytoplasm.

Cytokines are recognized as small and nonstructural proteins, which are pleiotropic and have multiple diverse biological activities [29]. Several studies have demonstrated that progesterone could modulate inflammatory cytokine production in vivo and vitro [30, 31]. As revealed by He et al. [17], post-TBI progesterone administration might attenuate the production of proinflammatory cytokines, such as IL-1β and TNF-α, in the injured brain. Another research [32] indicated that the abundant expression of TNF-α might be a molecular basis characteristic of leiomyomas in the human uterus and that progesterone might play a vital role in down-regulating the expression of TNF-α in human uterine leiomyoma. Our study showed that intestinal TNF-α and IL-1β levels were significantly induced by TBI at 5 days after trauma. Progesterone administration following TBI could down-regulate the expressions of TNF-α and IL-1β in the gut. However, it is still unclear that how progesterone modulates the inflammatory signals and the whole mechanisms involved call for further research.

In summary, to the best of our knowledge, this is the first study to demonstrate the effects of progesterone on the intestinal expression of inflammatory agents after TBI. We found that progesterone administration could suppress the TBI-induced NF-κB activation in the gut, decrease the intestinal production of proinflammatory cytokines, and protect ileum mucosa structure. These results suggest that the therapeutic benefit of post-TBI progesterone injections might be due to its inhibitory effects on NF-κB activity and proinflammatory cytokines expression in the small bowl. 

## Figures and Tables

**Table 1:** Changes in villous height, diameter, crypt depth, and surface area of mucosa (Values were expressed as mean ± SD.)

Groups	Villous height (*μ*m)	Villous diameter (*μ*m)	Crypt depth (*μ*m)	Surface area (mm^2^)
SV	294.1±41.8	63.0±15.5	80.2±12.5	0.0577±0.0155
LV	216.8±12.3*	54.2±2.0	55.2±1.8*	0.0370±0.0034
LP	257.5±40.3 ^##^	54.4±5.6	66.5±1.6 ^##^	0.0448±0.0053 ^#^

*P⁢< .05 versus SV group.

^#^ P⁢< .05 and ^##^ P⁢< .01 versus LV group.

## References

[1] Grotz MRW, Deitch EA, Ding J, Xu D, Huang Q, Regel G (1999). Intestinal cytokine response after gut ischemia: role of gut barrier failure. *Annals of Surgery*.

[2] Faries PL, Simon RJ, Martella AT, Lee MJ, Machiedo GW (1998). Intestinal permeability correlates with severity of injury in trauma patients. *The Journal of Trauma*.

[3] Swank GM, Deitch EA (1996). Role of the gut in multiple organ failure: bacterial translocation and permeability changes. *World Journal of Surgery*.

[4] Hang C-H, Shi J-X, Li J-S, Wu W, Yin H-X (2003). Alterations of intestinal mucosa structure and barrier function following traumatic brain injury in rats. *World Journal of Gastroenterology*.

[5] Hang C-H, Shi J-X, Li J-S, Li W-Q, Wu W (2005). Expressions of intestinal NF-κB, TNF-α, and IL-6 following traumatic brain injury in rats. *Journal of Surgical Research*.

[6] Chen F, Castranova V, Shi X (2001). New insights into the role of nuclear factor-κB in cell growth regulation. *American Journal of Pathology*.

[7] Castro-Caldas M, Mendes AF, Carvalho AP, Duarte CB, Lopes MC (2003). Dexamethasone prevents interleukin-1β-induced nuclear factor-κB activation by upregulating IκB-α synthesis, in lymphoblastic cells. *Mediators of inflammation*.

[8] Homaidan FR, Chakroun I, El-Sabban ME (2003). Regulation of nuclear factor-κB in intestinal epithelial cells in a cell model of inflammation. *Mediators of Inflammation*.

[9] Claud EC, Zhang X, Petrof EO, Sun J (2007). Developmentally regulated tumor necrosis factor-α induced nuclear factor κB activation in intestinal epithelium. *American Journal of Physiology*.

[10] Baeuerle PA, Baltimore D (1996). Nf-κB: ten years after. *Cell*.

[11] Lee JW, Lee MS, Kim TH (2007). Inhibitory effect of inflexinol on nitric oxide generation and iNOS expression via inhibition of NF-κB activation. *Mediators of Inflammation*.

[12] Fiocchi C (1997). Intestinal inflammation: a complex interplay of immune and nonimmune cell interactions. *American Journal of Physiology*.

[13] Pritts TA, Moon MR, Fischer JE, Salzman AL, Hasselgren P-O (1998). Nuclear factor-κB is activated in intestinal mucosa during endotoxemia. *Archives of Surgery*.

[14] Neurath MF, Pettersson S, Meyer zum Buschenfelde K-H, Strober W (1996). Local administration of antisense phosphorothioate oligonucleotides to the p65 subunit of NF-κB abrogates established experimental colitis in mice. *Nature Medicine*.

[15] Stein DG (2001). Brain damage, sex hormones and recovery: a new role for progesterone and estrogen?. *Trends in Neurosciences*.

[16] Wright DW, Kellermann AL, Hertzberg VS (2007). ProTECT: a randomized clinical trial of progesterone for acute traumatic brain injury. *Annals of Emergency Medicine*.

[17] He J, Evans C-O, Hoffman SW, Oyesiku NM, Stein DG (2004). Progesterone and allopregnanolone reduce inflammatory cytokines after traumatic brain injury. *Experimental Neurology*.

[18] Pettus EH, Wright DW, Stein DG, Hoffman SW (2005). Progesterone treatment inhibits the inflammatory agents that accompany traumatic brain injury. *Brain Research*.

[19] Feeney DM, Boyeson MG, Linn RT, Murraya HM, Daila WG (1981). Responses to cortical injury—I: methodology and local effects of contusions in the rat. *Brain Research*.

[20] Djebaili M, Hoffman SW, Stein DG (2004). Allopregnanolone and progesterone decrease cell death and cognitive deficits after a contusion of the rat pre-frontal cortex. *Neuroscience*.

[21] Goss CW, Hoffman SW, Stein DG (2003). Behavioral effects and anatomic correlates after brain injury: a progesterone dose-response study. *Pharmacology Biochemistry and Behavior*.

[22] De Bosscher K, Schmitz ML, Berghe WV, Plaisance S, Fiers W, Haegeman G (1997). Glucocorticoid-mediated repression of nuclear factor-κB-dependent transcription involves direct interference with transactivation. *Proceedings of the National Academy of Sciences of the United States of America*.

[23] Kalkhoven E, Wissink S, van der Saag PT, van der Burg B (1996). Negative interaction between the RelA(p65) subunit of NF-κB and the progesterone receptor. *The Journal of Biological Chemistry*.

[24] Allport VC, Slater DM, Newton R, Bennett PR (2000). NF-κB and AP-1 are required for cyclo-oxygenase 2 gene expression in amnion epithelial cell line (WISH). *Molecular Human Reproduction*.

[25] Allport VC, Pieber D, Slater DM, Newton R, White JO, Bennett PR (2001). Human labour is associated with nuclear factor-κB activity which mediates cyclo-oxygenase-2 expression and is involved with the ‘functional progesterone withdrawal’. *Molecular Human Reproduction*.

[26] Wissink S, van Heerde EC, van der Burg B, van der Saag PT (1998). A dual mechanism mediates repression of NF-κB activity by glucocorticoids. *Molecular Endocrinology*.

[27] Horie S, Harada T, Mitsunari M, Taniguchi F, Iwabe T, Terakawa N (2005). Progesterone and progestational compounds attenuate tumor necrosis factor α-induced interleukin-8 production via nuclear factor κB inactivation in endometriotic stromal cells. *Fertility and Sterility*.

[28] Davies S, Dai D, Feldman I, Pickett G, Leslie KK (2004). Identification of a novel mechanism of NF-κB inactivation by progesterone through progesterone receptors in Hec50co poorly differentiated endometrial cancer cells: induction of A20 and ABIN-2. *Gynecologic Oncology*.

[29] Sercombe R, Tran Dinh YR, Gomis P (2002). Cerebrovascular inflammation following subarachnoid hemorrhage. *The Japanese Journal of Pharmacology*.

[30] Gotkin JL, Celver J, McNutt P (2006). Progesterone reduces lipopolysaccharide induced interleukin-6 secretion in fetoplacental chorionic arteries, fractionated cord blood, and maternal mononuclear cells. *American Journal of Obstetrics and Gynecology*.

[31] Shields AD, Wright J, Paonessa DJ (2005). Progesterone modulation of inflammatory cytokine production in a fetoplacental artery explant model. *American Journal of Obstetrics and Gynecology*.

[32] Kurachi O, Matsuo H, Samoto T, Maruo T (2001). Tumor necrosis factor-α expression in human uterine leiomyoma and its down-regulation by progesterone. *The Journal of Clinical Endocrinology and Metabolism*.

